# *Ex vivo* biomechanical characterization of syringe-needle ejections for intracerebral cell delivery

**DOI:** 10.1038/s41598-018-27568-x

**Published:** 2018-06-15

**Authors:** Brendon Wahlberg, Harmanvir Ghuman, Jessie R. Liu, Michel Modo

**Affiliations:** 1 Departments of Radiology, University of Pittsburgh, Pittsburgh, USA; 2 Departments of Bioengineering, University of Pittsburgh, Pittsburgh, USA; 3 McGowan Institute for Regenerative Medicine, University of Pittsburgh, Pittsburgh, USA; 4 Centre for Neural Basis of Cognition, Pittsburgh, PA15203 USA

## Abstract

Intracerebral implantation of cell suspensions is finding its clinical translation with encouraging results in patients with stroke. However, the survival of cells in the brain remains poor. Although the biological potential of neural stem cells (NSCs) is widely documented, the biomechanical effects of delivering cells through a syringe-needle remain poorly understood. We here detailed the biomechanical forces (pressure, shear stress) that cells are exposed to during ejection through different sized needles (20G, 26G, 32G) and syringes (10, 50, 250 µL) at relevant flow rates (1, 5, 10 µL/min). A comparison of 3 vehicles, Phosphate Buffered Saline (PBS), Hypothermosol (HTS), and Pluronic, indicated that less viscous vehicles are favorable for suspension with a high cell volume fraction to minimize sedimentation. Higher suspension viscosity was associated with greater shear stress. Higher flow rates with viscous vehicle, such as HTS reduced viability by ~10% and also produced more apoptotic cells (28%). At 5 µL/min ejection using a 26G needle increased neuronal differentiation for PBS and HTS suspensions. These results reveal the biological impact of biomechanical forces in the cell delivery process. Appropriate engineering strategies can be considered to mitigate these effects to ensure the efficacious translation of this promising therapy.

## Introduction

The clinical potential of cell therapy is driven by the biological activity of cells in restoring, repairing or replacing lost cells/tissues. However, this potential can only be realized if cells are appropriately delivered^1^. The brain especially poses a delivery challenge due to its encasement by the skull and target sites often being seated deep below functional tissue. A minimally invasive implantation procedure is therefore required. This is commonly achieved through a needle attached to a syringe and requires injection of high-density cell preparations near sites of damage by applying external force. The safety of this intracerebral implantation of cells, as well as tissue pieces, has been demonstrated in phase I clinical trials with no major side effects from the procedure^2–4^. Nevertheless, the survival of cells using this procedure indicates a poor retention and survival of cells. Cell retention/survival rates of approximately 5% of implanted cells are reported^5^. While the inflammatory host microenvironment around the damaged tissue may affect the survival after transplantation, cell damage may first occur during injection from the shear mechanical forces inside the needle-syringe assembly. Delivery of cells is therefore a key process to ensure efficacy of intracerebral stem cell implantation^1^.

Cell delivery through a needle-syringe is achieved by suspending cells in a liquid phase vehicle. The process of suspending cells can affect their viability and affect cell clumping, as well as sedimentation^6^. The biophysical properties of the suspension vehicle and cells, such as viscosity and density, interact with the syringe-needle design characteristics to determine the biomechanical forces generated by the ejection procedure. The viscosity of the suspension vehicles determines shear stress and influences the force required for ejection^7,8^. Wall shear stress affects cell function, such as the secretion of pro-inflammatory cytokines from mesenchymal stem cells (MSCs)^9^. In addition to the suspension vehicle and bore size, wall shear stress is modulated through the applied force to eject cells. This applied force is defined by the ejection parameters, such as the speed of ejection (also known as flow rate). Ejection parameters have been shown to affect viability of cells^10–12^. Importantly, intravenous (i.v.) and intra-arterial (i.a.) injections are into an aqueous solution (i.e. blood), whereas intracerebral injections are typically into the brain parenchyma that acts as a solid or semi-solid. Significant differences in flow/ejection rates are therefore being used for i.v. or i.a. delivery of cells through catheters (400–1200 µL/min)^11^ compared to intracerebral syringe-needle injections (1–10 µL/min)^3,4^. Using MSCs, it has been shown that smaller needle bore size increases apoptosis in ejected cells^13^. A slower flow rate attenuates this effect^8^. To avoid the deleterious effects of the ejection process of cells for tissue injection, it is hence essential to characterize the biomechanical forces cells are exposed to during a syringe-needle injection and to define optimal parameters.

Although extensive work on the intracerebral delivery of fetal tissue pieces has been performed, little work has been done on human neural stem cells (NSCs) in cell suspensions for intracerebral injection^3^. To evaluate these biomechanical forces on NSCs, we here measured the ejection pressure for different syringe (10, 50, 250 µL) and needle (20G, 26G, 32G) combinations and compared 3 common suspension vehicles (phosphate buffered saline, HypoThermosol, Pluronic F68) using different flow/ejection rates (1, 5, 10 µL/min). To determine the biological effects of these conditions, cell viability, cell membrane damage, apoptosis, and cell differentiation were measured. Based on these investigations, optimal parameters for cell delivery can be determined.

## Methods

### Suspension Vehicles

A solution buffer consisting of phosphate buffered saline (0.01 M PBS, P4417, Sigma-Aldrich), a cryopreservation solution HypoThermosol (HTS, H4416, Sigma-Aldrich), and Pluronic F68 (P1300, Sigma-Aldrich) were used as vehicles for cell suspension. We have previously reported the density (PBS 1.02 g/mL; HTS 0.98 g/mL; Pluronic 0.97 g/mL) and viscosity (PBS 0.92 cp; HTS 3.39 cp; Pluronic 0.99 cp) measurements for these solutions, as well as their performance to maintain cell suspensions^6^. All solutions were sterilized using a 0.2 µm filter prior to use.

### Syringe-needle preparation

**Figure 1:**
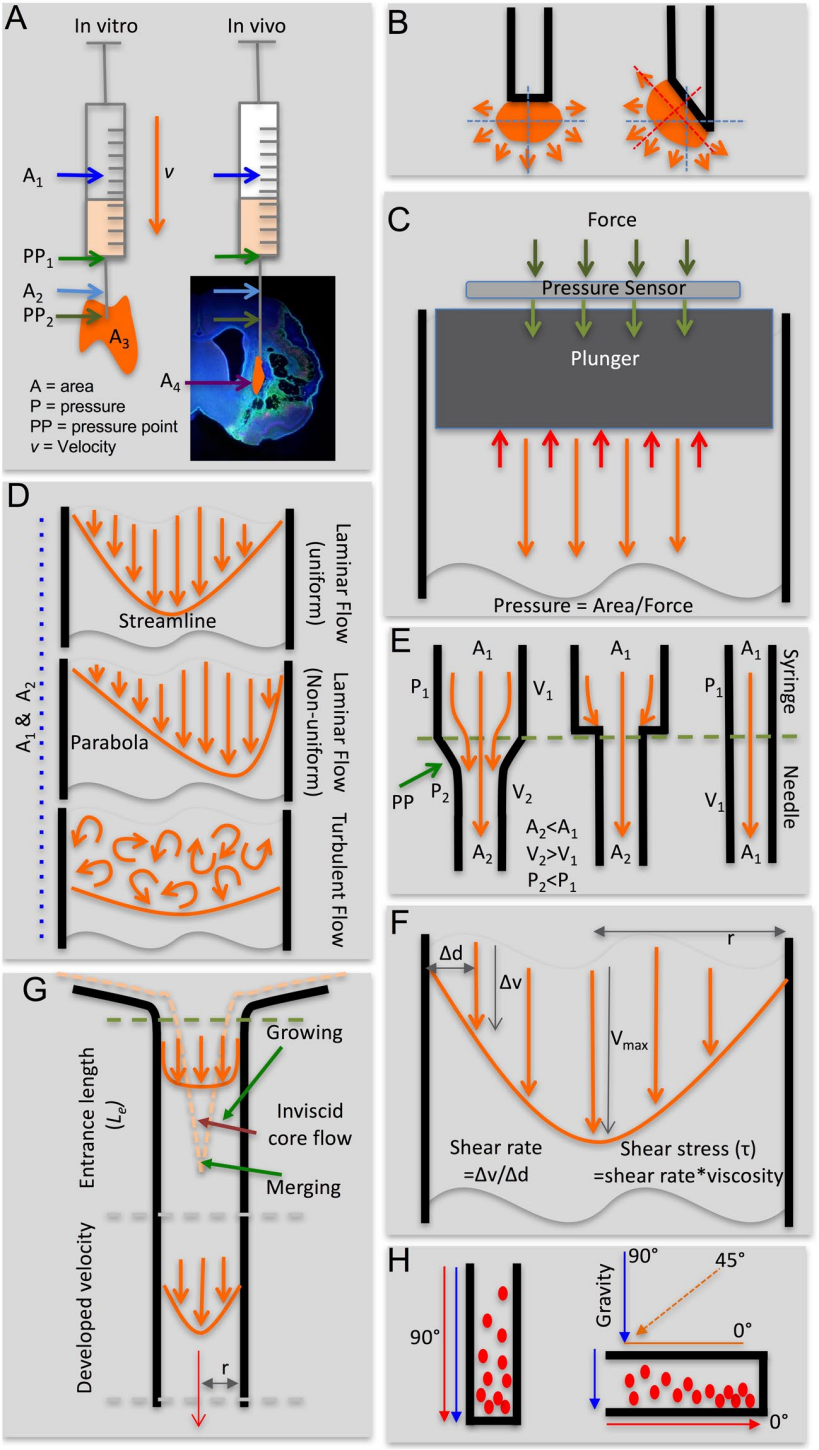
Biophysical and biomechanical considerations for syringe-needle ejections. (**A**) The ejection pressure from needle-syringe is defined by the area of the barrel and the velocity (v) to move the plunger. The area of the barrel inside the syringe (A_1_) can be of a different size than inside the needle (A_2_). Ejection *in vitro* into an empty space (A_3_) versus in tissue *in vivo* (A_4_) further influences the force required to push the plunger at a given velocity. As the area of the ejectate changes between the syringe and the needle, as well as the needle and the environment, pressure points (PP) are formed. (**B**) The point of ejection is defined by the shape of the needle tip being flat or beveled, which will influence the dispersion of the ejectate. (**C**) Pressure within the syringe and needle barrel was calculated based on the measurement of the applied force using a pressure sensor placed onto of the plunger. (**D**) Based on Reynold’s numbers, the flow characteristics within the barrel were defined to be uniform or non-uniform laminar flow or turbulent flow. (**E**) The interface between syringe and needle defined pressure points and potentially affects flow characteristics. A straight barrel between syringe and needle is the most optimal arrangement to avoid pressure points as well as to minimize the formation of a plug that could block the ejection. (**F**) Within the syringe and needle, shear rate and stress can be calculated based on the radius of the barrel, the viscosity of the material, and the flow rate. (**G**) During the transition between the syringe and the needle, the suspension will comply with an entrance length (L_e_) which will allow the streamline to move along the barrel and develop velocity. (**H**) Cells in the suspension along the path of ejection will sediment if their density is higher than the vehicle. The sedimentation rate is dependent on the angle of the barrel with an orientation along the path of gravity (90°) exerting maximal sedimentation and least sediment along the barrel being observed with a horizontal orientation (0°).

For intracerebral implantation of cells, the choice of syringe is dependent on the volume of injection, with a 10 µL Hamilton syringe^5^ being most commonly used in preclinical studies, whereas 50 to 250 µL syringes were used in recent clinical trials^3,4^. With respect to biomechanical forces, it is important to note that these syringes have different bore sizes (Table 1) that affect shear stress. The barrel diameter defines the area (A) through which the cell suspension traverses at a given velocity (*v*) to create a pressure point (PP), exerting biomechanical effects on cells as they are ejected (Fig. 1A). Barrel length is also different between 10 and >50 µL syringes, which can affect sedimentation. To cover a range of different volumes, we here included 10, 50 and 250 µL Hamilton syringes. The bore size of needles also varies and affects the exposure of cells to shear stress, as well as dead volume (Table 2). Larger bore sizes (20G) are expected to induce less shear stress than smaller bore sizes (32G). It is also important to consider cellular throughput. A large 20G needle (0.603 mm diameter) can pass <31 cells (at a cell diameter of 19.29 µm), with a 32G needle (0.108 mm) being even more limiting with <5 cells side-by-side fitting through the needle. However, consideration also needs to be given to tissue damage caused by needle penetration and a medium size needle (26G) might therefore provide optimal conditions balancing *in vitro* and *in vivo* performance characteristics. Only blunt metal needles (point 2 style) were used here, as they minimize the tissue damage during insertion and provide a bolus of ejection that distributes equally in all directions (Fig. 1B).

**Table 1:** Physical Properties of Syringes.

Volume (μL)	Model	Diameter		Length (mm)
		Outer (mm)	Inner (mm)	
5	7634–01	6.604	0.343	54.1
10	7635–01	6.604	0.485	54.1
25	7636-01	7.747	0.729	60
50	7637-01	7.747	1.030	60
100	7638-01	7.747	1.457	60
250	7639-01	7.747	2.304	60
500	7640-01	7.747	3.256	60

**Table 2:** Physical Properties of Needles.

Gauge	Model	Length (mm)	Diameter		Wall (mm)	Dead Volume (μL)
			Outer (mm)	Inner (mm)		
18	7804-06	50	1.279	0.838	0.430	110.25
20	7804-11	50	0.908	0.603	0.300	57.08
22	7804-01	50	0.718	0.413	0.300	26.77
24	7804-08	50	0.566	0.311	0.250	15.18
26	7804-03	50	0.464	0.260	0.200	10.61
32	7804-04	50	0.235	0.108	0.064	1.83

Syringes were cleaned using Hamilton cleaning solution (Hamilton) followed by pressurized air to remove all fluid from the syringe barrel. Syringes were sterilized by UV irradiation overnight. Plungers were placed in an alcohol solution followed by a sterile PBS wash and UV irradiation as above. Needle interiors were cleaned by drawing up and washing with sterile water followed by sterile PBS, and were sterilized in a Germinator hot bead dry sterilizer (CellPoint Scientific) for 5 minutes. At present, no performance criteria are found in the literature regarding the repeat use of syringes and needles. We noted that new needles require very little force for the plunger to drop in a vertical position (Supplementary Fig. 1). Without cleaning there is a gradual build-up of resistance due to friction within the syringe that is mostly mitigated by cleaning. The use of vehicles further reduces this strain, but still requires more force than a new syringe. We here implemented the performance criteria that new or cleaned syringes require no more than 0.2 N in ejection force (which correspond to 1.1 and 3.77 MPa in ejection pressure for a 10 μL syringe and 26G needle) without vehicle being replaced. This criterion served as the minimum ejection pressure required to overcome the frictional resistance in the syringe wall-plunger interface.

### Ejection pressure measurement

To measure ejection pressure, syringe-needles were mounted vertically (90°) on a stereotactic frame (Kopf). An LCKD-1KG series subminiature compression load cell (Omega) was positioned on top of the syringe plunger (Fig. 1C). A high-performance strain gage indicator (DP41-S, Omega) was zeroed prior to recording applied force (mN) every 10 seconds (Supplementary Fig. 2 charts maximum force measurements). To standardize measurements, a total volume of 10 μL was ejected for all conditions. Injection speed (1, 5, 10 μL/min) was controlled by a Micro4 microsyringe pump controller (World Precision Instruments). The pressure (Pa) in the suspension vehicle was measured using the following formula:

1 $$Pressure=\frac{Force}{Area}$$

Normal intracranial pressure (ICP) is considered 7–15 mmHg (equivalent to 0.93–1.99 kPa). ICP measurements >25 mmHg (3.33 kPa) typically require an intervention to reduce pressure^14^. Ejection pressures >3.33 kPA are therefore here considered atypical of the normal brain environment.

### Flow characteristics - Reynolds number

Injection speed and diameter of the barrel influence the flow characteristics of the cell suspension. Laminar flow provides a uniform streamline of passage through the barrel characterized by Reynolds (Re) numbers <2,100 (Fig. 1D). A uniform laminar flow is defined by Re < 0.1. In contrast, turbulent flow occurs with Re >4,000. Transitional non-uniform flow occurs between 2,100–4,000 Re. The Reynolds number for each condition was calculated using the following formula to determine flow characteristics:

2 $$Re=\frac{\rho Q}{15\pi D\eta }$$

where *ρ* is the density of the vehicle (g/μL), *Q* is volumetric flow rate (μL/s), *D* is the diameter of either the needle or syringe (cm), and *η* is dynamic viscosity of the vehicle (kg/(m s)). Syringe-needle combinations where bore size remains the same have a homogenous flow between both compartments, as the transition zone between syringe and needle does not create a pressure point (Fig. 1E). However, most flexible systems with removal needles will have a mismatch in bore size and pressure inside the needle will be higher. These design characteristics can affect flow and lead to changes in shear rate and stress.

### Shear Stress - Law of Poiseuille

As the cell suspension flows through the syringe-needle barrel, it encounters the barrel wall which is immobile. The shearing force caused by interaction with the barrel wall produces a deformation of the cell suspension defined as the shear rate (Fig. 1F). The shear rate is lower with inviscid core flow at the center of the barrel (Fig. 1G) and highest at the edge of the wall. The parabolic velocity profile at the core of the suspension hence flows faster than the edge. The flow rate and the viscosity of the suspension determine shear stress. The wall shear stress for each condition was calculated in both the needle and the syringe for various flow rates according to the Law of Poiseuille:

3 $${\tau }_{max}=\frac{4Q\eta }{\pi {R}^{3}}$$

where *τ*_*max*_ is shear stress (N/m^2^), *Q* is volumetric flow rate (cm^3^/s), *η* is dynamic viscosity of the vehicle (kg/(m s)), and *R* is the inner radius of either the needle or syringe (m). Physiological shear stress in human arteries ranges from 1–7 N/m^2^ and in the venous system from 0.1–0.6 N/m^2 ^^15,16^. In rodents, this can be up to an order of magnitude higher^17,18^. However, experiments in cortical neurons indicate that shear stress as low as 1 N/m^2^ for 1 hour can significantly reduce cell viability, especially with a prolonged exposure^19^. A shear stress of 0.5 N/m^2^ has been demonstrated to exert no significant effect on cortical neuron viability^19^ and is therefore here considered as threshold to define acceptable shear stress conditions.

### Neural Stem Cells

The human cortical neural stem cell (NSC) line CTX0E03 (ReNeuron, UK) was cultured, as previously described in detail^20^. In brief, the cell line was derived from the cortical region of a human fetus 12 weeks old and conditionally immortalized using cMyc-ER^TAM^ under the control of 4-hydroxytamoxifen (4-OHT, 100 nM, Sigma). In the absence of 4-OHT, NSCs will cease proliferation and undergo differentiation. NSCs were expanded on laminin (10 µg/mL, Sigma) coated flasks until 80–85% confluency was reached. Recombinant human basic fibroblast growth factor (bFGF, 10 ng/mL, PeproTech) and epidermal growth factor (EGF, 20 ng/mL, PeproTech) were used as mitogens (Table 3 for complete description of media components). All culturing was performed without the addition of antibiotics at 37 °C in 5% CO_2_. Cells were tested monthly for mycoplasma using a PCR kit (Sigma) or colorimetric analysis (Plasmotest, Invitrogen) and found to be negative. A biophysical characterization of NSCs indicated an average cell diameter of 19.29 µm, yielding a cell volume of 3,912 µm^3^ and a density of 2.04 g/mL^6^.

**Table 3:** Components added to DMEM-F12 basal medium for STROC05 proliferation medium.

Component	Source	Final Concentration
Human Albumin Solution	GemBio 800–121	0.03%
Transferrin, human	Sigma T1147	100 µg/ml
Putrescine DiHCl	Sigma P5780	16.2 µg/mL
Insulin, human recomb.	Sigma I9278	5 µg/mL
L-Thyroxine (T4)	Sigma T0397	400 ng/mL
Tri-iodo-thyronine (T3)	Sigma T6397	337 ng/mL
Progesterone	Sigma P8783	60 ng/mL
L-glutamine	Sigma G7513	2 mM
Sodium Selenite	Sigma S9133	40 ng/mL
Heparin Sodium	Sigma H3149	10 units/mL
Corticosterone	Sigma C2505	40 ng/mL
bFGF	PeproTech 100-18B	10 ng/mL
EGF	PeproTech AF-100-15	20 ng/mL
4-hydroxy-tamoxifen (4-OHT)	Sigma H7904	100 nM

For differentiation medium, bFGF, EGF and 4-hydroxy-tamoxifen (shaded area) were omitted.

### Preparation of cell suspension

At 80–85% confluency, cells were harvested from their flask after adding 5 mL of Accutase in a T75 flask and counted, as previously described^6^. Cells were spun down with supernatant aspirated to afford suspension in a given vehicle at a 50,000 cells/µL concentration (~20% volume fraction). The following formula was used to calculate the appropriate volume of suspension vehicle to be added to the cell pellet to yield a given concentration/volume fraction:

4 $${V}_{V}={V}_{T}-{V}_{C}$$

where V_V_ = volume of vehicle to be added; V_T_ = total volume of preparation; V_C_ = total cell volume.

### Cell volume fraction and suspension density

The density of a vehicle is an important measure that influences buoyancy, as well as sedimentation. The density of vehicles was determined by weighing 1 mL of each vehicle on a precision scale (Ohaus, Parsipanny, NJ, USA). The weight of the vehicle (in grams) was then divided by the total volume (i.e. 1 mL) to obtain density in g/mL. In order to measure the suspension density of different cell volume fractions, the mass of a single cell was first measured by weighing 10^4^, 10^5^ and 10^6^ cells suspended in 10 μL of PBS and then subtracting the weight of 10 μL PBS alone. Once the mass of a single cell is determined, the total mass of a cell suspension can then be calculated by adding the mass of total cells in a known cell fraction (20% cell fraction in 1 mL suspension requires 5 × 10^7^ cells) with the mass of vehicle alone. The combined mass was then divided by the total volume of suspension (1 mL) to yield the suspension density of different theoretical volume fractions. According to the random closed packing (RCP) paradigm, spheres of equal size can achieve a maximum volume fraction of 0.636^21^, indicating that practically a ~60% volume fraction is the highest achievable cell content and equivalent to a cell pellet.

### Viscosity of cell suspensions

The viscosity of cell suspensions is defined by both vehicles and cells. Their relative volume fraction defines their relative weight to the overall viscosity of the suspension. The viscosity of the suspension can be calculated for different cellular fractions according to the Krieger-Dougherty equation:

5 $${\eta }_{s}={\eta }_{v}\ast {(1-\frac{\varphi }{{\varphi }_{m}})}^{-{\eta }_{i}{\varphi }_{m}}$$

where *η*_*s*_ (cP) is the dynamic viscosity of the suspension, *η*_*v*_ (cP) is the dynamic viscosity of the vehicle, *φ* is the volume fraction of the cells in the suspension, *φ*_*m*_ is the maximum volume fraction of the cells in the suspension, and *η*_*i*_ is the intrinsic viscosity of the medium. For spherical particles such as the cells in suspension, *η*_*i*_ is 2.5. Additionally, *φ*_*m*_ was defined as 1 for a suspension with no vehicle. Further, *φ* was calculated by dividing the target cell concentration (5 × 10^4^, 1 × 10^5^, 1.5 × 10^5^, 2 × 10^5^) by the maximum concentration of cells (2.5 × 10^5^ cells/μL).

### Cell sedimentation rate

Cells in suspension can either sediment due to the influence of the gravitation force (*F*_*g*_) or lift due to the buoyancy force (*F*_*b*_), which opposes *F*_*g*_. The drag force (*F*_*d*_) describes the fluids resistance to the cells movement. In vehicles with a density lower than cells (ρ < 2.04 g/mL), the gravitational force acts on cells to sediment. However, the angle of the barrel affects the sedimentation rate (Fig. 1H). The sedimentation rate of cells was calculated for three different angles of the syringe during injections (1° horizontal position, 45° angle, 90° vertical position) using the following formula:

6 $${v}_{s}=\frac{{d}^{2}({\rho }_{c}-{\rho }_{v})g}{18\eta }$$

where *v*_*s*_ is the sedimentation rate (μm/s), *d* is the diameter of the cell (m), *ρ*_*c*_ is the density of the cell (kg m^−3^), *ρ*_*v*_ is density of the vehicle (kg m^−3^), *g* is gravitational acceleration at each angle of injection (m s^−2^), and *η* is the dynamic viscosity of the vehicle (kg(m s)).

### Drag force at cell-vehicle interface – Stoke’s law

The mean velocity of cell suspension passing through a syringe or needle under a constant flow rate can be calculated using the following equation:

7 $$v=\frac{Q}{\pi {R}^{2}}$$

where *v* is the mean suspension velocity inside a syringe or needle (m/s), *Q* is volumetric flow rate (m^3^ s^−1^), and *R* is the inner radius of either the needle or syringe (m). The velocity of the cells moving through a fluid influences the drag force (*F*_*d*_) that cells are exposed to. Objects with very small Reynolds numbers (Re < 0.1) in viscous fluids are exposed to frictional *F*_*d*_ that can be described by Stoke’s law. The Stokes drag force exerted on cells can be calculated using Stoke’s formula for *F*_*d*_ that is acting at the interface between the fluid and cells:

8 $${F}_{d}=6\pi \eta rv$$

where *F*_*d*_ is the drag force (N), *η* is dynamic viscosity of the vehicle (Pa s), *r* is the radius of the cell (m) and *v* is flow velocity.

### Cell viability

To determine effects of vehicle suspension, ejection speed and needle size on cell viability, NSCs were suspended in PBS, HTS, or Pluronic at a concentration of 50,000 cells/μL (20% volume fraction) and loaded into a 10 μL Hamilton syringe (500,000 cells total per individual replicate) with either a 26G or 32G needle attached and ejected at 1, 5, 10 μL/min. A 1 μL volume of cells was ejected into a 0.5 mL Eppendorf tube containing 5 μL of PBS. 5 μL were taken up into a P10 pipette and added to a hemocyotmeter (Fisher). To distinguish dead from live cells, a 6 μL volume of trypan blue (Sigma) was added to visualize dead cells.

### Cell membrane damage

The effect of mechanical stress on cell damage was investigated using lactate dehydrogenase (LDH) release (TOX7, Sigma)^6,22^. For this, a 1 μL volume (20% volume fraction, 50,000 cells/μL) out of a 10 μL cell suspension (500,000 cells total per biological replicate) was ejected onto 13 mm circular coverslips (Gold Seal) coated with laminin containing proliferation media. A control condition consisted of ejecting 1 μL of cells from a P10 pipette (Eppendorf) using a 10 μL tip (Fisher SureOne). The 24-well plates were incubated overnight, and 24 hours after injection, supernatants were collected from each well. Triplicate biological samples were analyzed using the LDH Cytotoxicity Assay Kit (Pierce). Results were read using a microplate reader (BioTek) at 490 and 680 nm. Absorbance at 490 nm for each experimental condition was compared to absorbance for samples from wells that had total lysis (100% LDH release).

### Cell apoptosis

At 24 hours post-ejection, NSCs seeded on coverslips (as per above) were fixed with 4% paraformaldehyde and assayed for apoptosis using immunohistochemistry. A primary antibody consisting of a rabbit anti-Caspase-3 (1:50; Millipore; AB3623) in PBS + 0.1% Triton X-100 was incubated with cells overnight at 4 °C followed by a goat anti-rabbit IgG Alexa Fluor 555 (1:500; ThermoFisher) secondary antibody for 1 hour at room temperature. To visualize individual nuclei for cell counting, Hoechst 33342 (1 μg/mL; Sigma) was applied as a counterstain for 5 min prior to placing the coverslip on a microscopic slide using Vectashield for fluorescence (ThermoFisher). Using a Zeiss AxioImager M2 Fluorescence Microscope, fifteen randomly chosen images (20x magnification) were acquired. For each condition, 45 images were analyzed using Fiji version 1.49 (https://fiji.sc).

### Cell differentiation

After 7 days of differentiation, NSCs (20% volume fraction, 50,000 cells/μL) were fixed using 4% paraformaldehyde to investigate if these different ejection conditions influence the differentiation phenotype of NSCs. Primary antibody staining was performed overnight at 4 °C with rabbit anti-Fox3 (1:500; Abcam ab177487) to measure neuronal differentiation and mouse anti-human Stem123 (1:1,000; Cellartis Y40420) to visualize astrocytes. A goat anti-mouse IgG Alexa Fluor 488 (1:500), and also goat anti-rabbit IgG Alexa Fluor 555 (1:500) secondary antibody was applied for 1 hour at room temperature before application of Hoechst 33342 to visualize individual cell nuclei. Cell counting was performed using 45 images acquired at 20x magnification using Fiji.

### Statistical analyses

Graphing was performed in Prism version 7 (GraphPad). Statistical analyses and contour plots were calculated in Minitab version 17 (Minitab). One-way analyses of variance (ANOVA) compared multiple conditions for a single independent and dependent variable (e.g. cell viability, LDH, cell differentiation) with Bonferroni post-hoc testing. Two-way ANOVAs were used to determine statistical differences between more than 1 independent variable (e.g. ejection pressure) using Tukey’s post-hoc test. A p value of <0.05 was considered significant. For contour plots, dependent variables were plotted against independent variables in 5–12 equal data ranges (bin sizes) to define contour lines that span the measured values and provide an overview of the interaction of independent variables on the dependent variables.

## Results

### Faster injections using low viscosity vehicles with larger syringes and needles reduce ejection pressure

**Figure 2:**
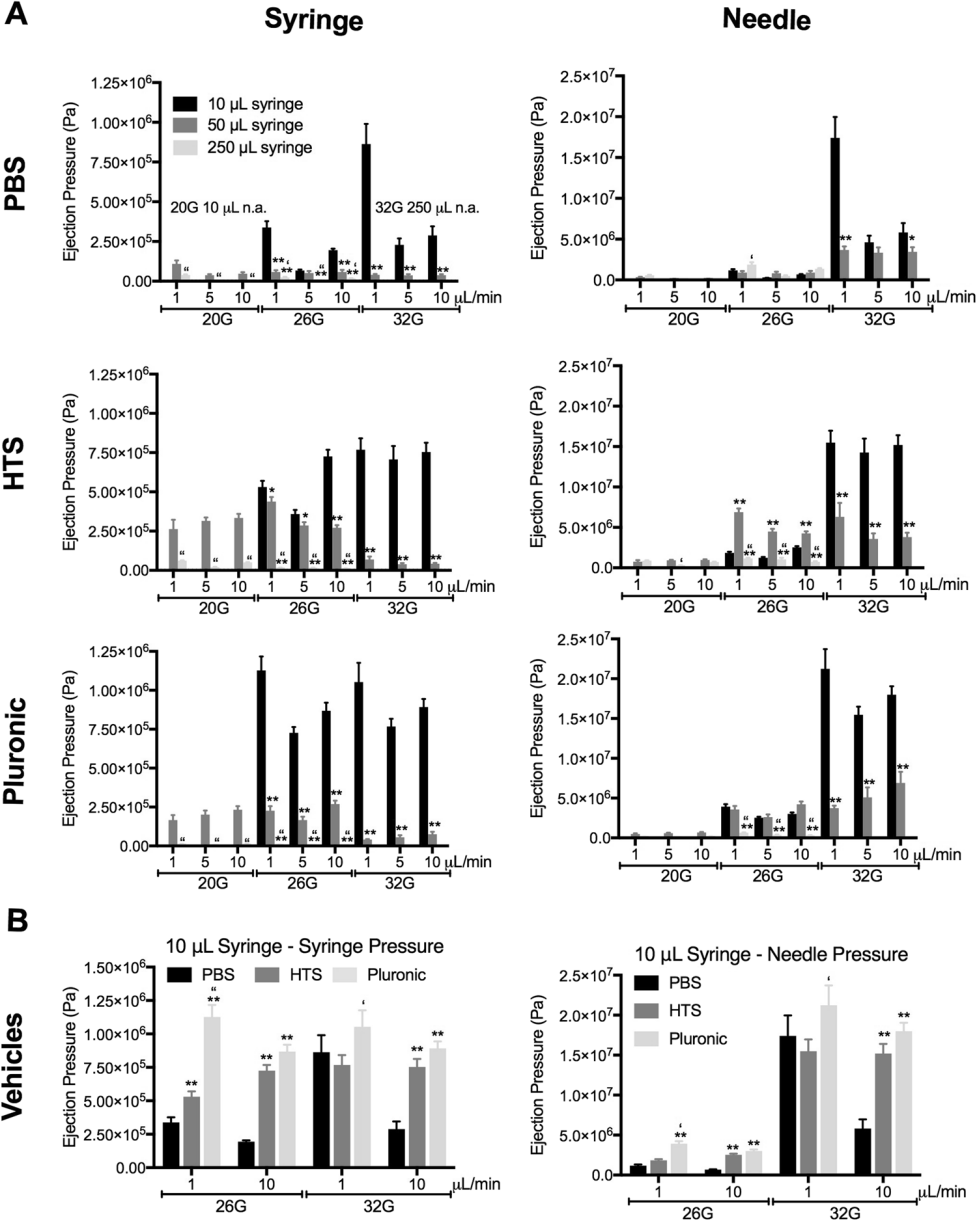
Syringe and needle pressures of suspension vehicle ejections. (**A**) Ejection pressure was calculated based on the measured force applied to eject phosphate buffered saline (PBS), hypothermosol (HTS) or pluronic at defined flow rates using different syringe-needle combinations. (**B**) A direction comparison of ejection pressures for the 3 suspension vehicles for syringe and needle combinations illustrates that faster ejection using large bore needle and less viscous vehicles reduces ejection pressure.

The ejection pressure in syringes and needles was calculated based on the measured force that was applied to eject PBS, HTS and Pluronic at speeds of 1, 5 and 10 μL/min (Fig. 2A). A maximum ejection pressure of 1.0528 × 10^6^ Pa (i.e. 1.05 MPa) was evident for Pluronic at a speed of 1 μL/min from a 10 μL syringe with a 32G needle. In contrast, ejection pressure was up to 20x higher in needles compared to syringes. The smaller bore size of needles produced significantly higher ejection pressures with a maximum ejection pressure of 2.12316 × 10^7^ Pa (21.23 MPa) for the 1 μL/min speed of pluronic from a 10 μL syringe with a 32G needle condition. The smallest 32G needle consistently produced the highest ejection pressure for all vehicles. A 7.3-fold increase in ejection pressure was evident between the 20G and 26G needle and a further 5.4-fold increase between a 26G and 32G needle for pluronic. Additional pressure increases were evident when the smaller needles were combined with smaller syringes. The least ejection pressure was present in the larger syringe (250 μL) with a large diameter needle (20G) at a 5 and 10 μL/min ejection speed. All conditions exceed normal levels of intracranial pressure (~2 kPA) by at least 50%. The only condition where ejection pressure did not exceed the level of intracranial pressure that would require treatment (3.3 kPA)  utilized PBS with an ejection speed of 5 μL/min in a 250 μL syringe and 20G needle (3.238 kPa). However, even under these conditions the maximum pressure inside the needle exceeded intracranial pressure 15 × (47.272 kPa). A direct comparison of vehicles in a 10 μL syringe, which is the most commonly used size for preclinical studies, indicated that PBS as vehicle produced the least ejection pressure and that faster speed of ejection also lowered ejection pressure (Fig. 2B). Ejection pressure in a 32G needle was significantly 6x higher than in a 26G needle at both 1 and 10 μL/min flow rate. It is important to note that we here measured maximum ejection pressure and that cells are only exposed to this for a brief time during uptake and ejection, which would be a maximum of 10 min at a slow delivery speed of 1 μL/min.

Contour plots of these variables further illustrate that faster injections with larger syringes and needles reduce ejection pressure and that less viscous vehicles, such as PBS, are also favorable for ejection (Supplementary Fig. 3A). A speed of 5 μL/min consistently reduces ejection pressure in syringes for all tested vehicles. Pressure inside the needles further indicates differences between vehicles that is dependent on the diameter of the needle (Supplementary Fig. 3B). The larger the diameter of the needle, the less ejection pressure for different vehicles. It is important to note here that the syringe and needle exert combined effects on ejection pressure. It is, for instance, noticeable that all ejection pressures for a 20G needle are lower when combined with a 250 μL syringe rather than a 50 μL syringe. This combined cross-over effect is especially apparent in the HTS condition where a 50 μL syringe produced the highest ejection pressure. A 5 μL/min flow rate in a 50 μL syringe combined with a 26G needle provides an optimal condition for all tested vehicles.

### Syringe-needle flow is a uniform laminar streamline

The flow characteristics through a tube are defined by the density, viscosity and flow rate of the vehicles, as well as the diameter of the tube. Flow rate and syringe-needle diameter define the speed at which the vehicle is moving through the barrel (Table 4). Different flow characteristics in the syringe and needle can therefore be expected if their diameter is different. The highest Reynolds number is achieved using pluronic at a flow rate of 10 μL/min in a 32G needle with a maximum Re of 0.03 (Supplementary Fig. 4A). This is well below the criterion for pure laminar flow at Re 0.1, indicating that all flow in these injection paradigms follows the characteristics of a pure laminar flow.

**Table 4:** Flow velocity of vehicle during ejection.

	Size	Flow rate (μL/min)	Flow velocity (μm/s)
Syringe	10 μL	1	90.25
		5	451.16
		10	902.54
	50 μL	1	20.02
		5	100.06
		10	200.17
	250 μL	1	4
		5	20
		10	40
Needle	20G	1	58.41
		5	291.98
		10	584.09
	26G	1	314.11
		5	1570.19
		10	3141.13
	32G	1	1820.66
		5	9101.14
		10	18206.64

### Shear stress is low, but increased with a smaller needle diameter and HTS

The viscosity of the suspension vehicle dramatically influences its interaction with the barrel wall of the syringe and needle. As HTS has the highest viscosity of the tested suspension vehicles, it resulted in the highest shear stress at 4 N/m^2^ with a 32G needle at 10 μL/min (Supplementary Fig. 4B). Lower flow rates reduce shear stress with minimal shear stress evident at rates <5 μL/min. Overall, low shear stress was observed that does not exceed 0.05 N/m^2^ for syringes and yielded <0.5 N/m^2^ within needles. The use of a 26G needle hence could reduce the higher shear stress observed with HTS, or alternatively pluronic or PBS could be used with a 32G needle to reduce shear stress if a very small needle bore is required.

### Vehicle viscosity and cellular fraction determine suspension viscosity and cell sedimentation

**Figure 3:**
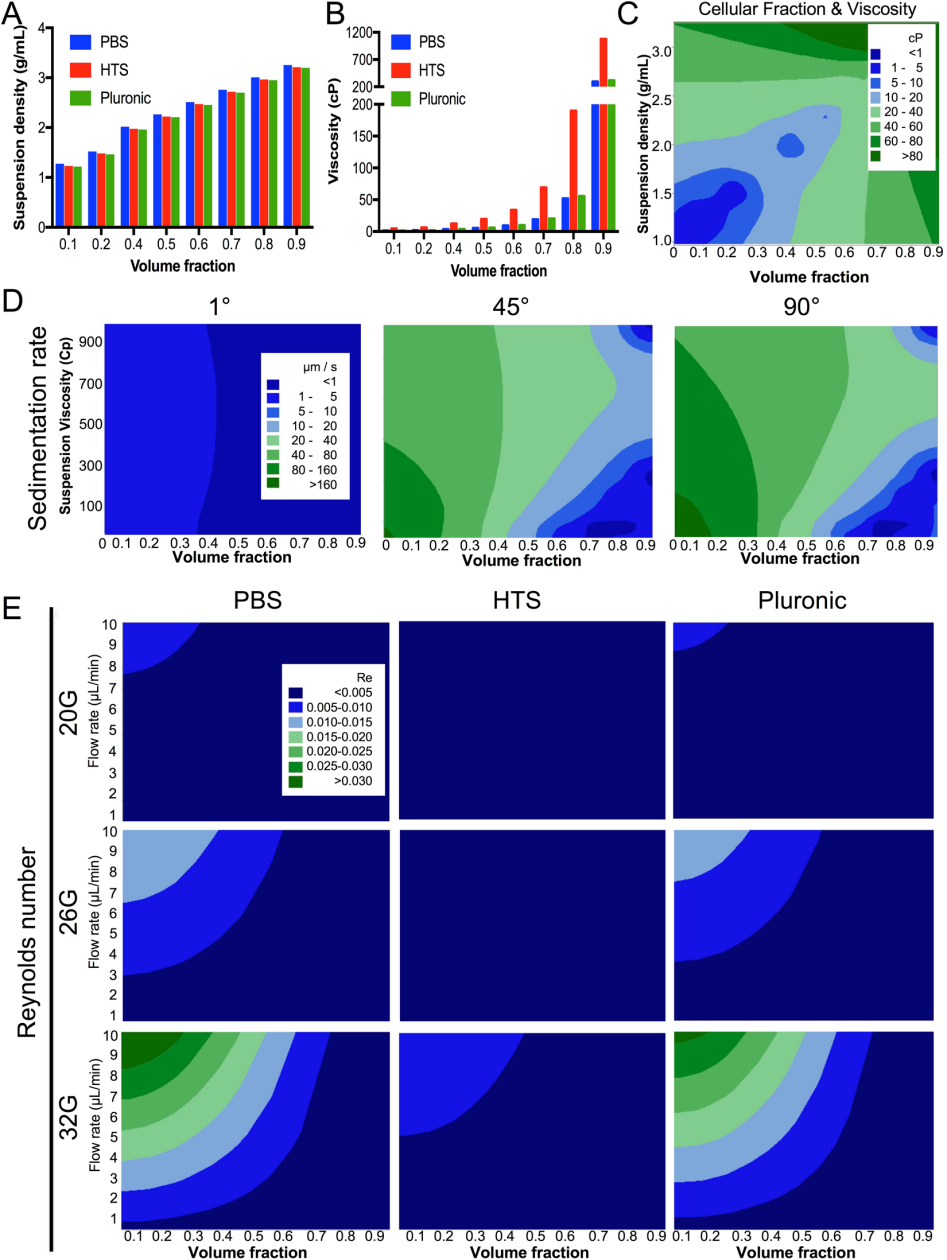
Cell suspension viscosity, sedimentation and Reynold’s numbers. (**A**) The density of cell suspensions is dependent on the volume fractions of cells and suspension vehicle. However, practically a ~60% volume fraction (150,000 cells/μL) is the highest achievable concentration due to the maximum packing density of spheres being 0.636. As the density of cells is higher than vehicles, increased volume fraction defined suspension density. (**B**) The viscosity of the cell suspension is also related to volume fraction. An exponential increase in viscosity is seen with increased cell volume fraction. (**C**) A contour plot further highlights the interaction between volume fraction and fluid density to define the viscosity of the cell suspension. (**D**) Sedimentation of cells at a horizontal orientation is minimal, whereas a significant sedimentation is seen at a vertical orientation with a speed of sedimentation >160 μm/s, if a 10% cell volume fraction (25,000 cells/μL) is used. Higher cell volume fractions reduce sedimentation rate. (**E**) Reynolds numbers for cell suspensions inside the needle remain well below the Re < 0.1 threshold to indicate that these would still flow in a uniform laminar streamline.

Cellular fraction affects the density of the cell suspension in a linear fashion with a 90% cell fraction producing a fluid density of ~3 g/mL (Fig. 3A). A more dramatic effect of cell fraction is observed on suspension viscosity which increases exponentially (Fig. 3B). Commonly used cell concentration of 50,000 cells/μL (20% cell fraction) and 100,000 cells/μL (40%) have significantly different suspension viscosities that can affect the viability of cells due to shear stress during ejection. Viscosity doubles between a 20% and 40% cell fraction for all suspension vehicles. However, with greater cell fractions the differences in viscosity between suspension vehicle become more important. For instance, the maximum packing density of suspended sphere/cells that can be achieved is ~60%. At this high cell density, PBS and pluronic have a viscosity 4 times lower than HTS. Fluid density and volume fraction act in a predictable synergistic fashion to increase suspension viscosity (Fig. 3C), providing a basis to adjust suspension vehicle properties to achieve a defined viscosity for a desired cell concentration. Higher packing densities might therefore benefit from the use of lower viscosity suspension vehicles, whereas lower packing density can benefit from more viscous materials.

Cell volume fraction and viscosity determine the rate of cell sedimentation in the suspension. However, sedimentation rate is also influenced by the angle at which the suspension is kept for ejection (Fig. 3D). At an almost horizontal angle (1°), cells undergo very little sedimentation along the barrel at speeds <5 μm/s, regardless of suspension viscosity. In contrast, at a vertical angle (90°), suspension viscosity and cell fraction interact to produce a high sedimentation of cells (>80 μm/s) if these are suspended at a low volume fraction (10%) and at a viscosity <500 cP. Sedimentation rate of cells can hence be influenced by choosing an appropriate suspension vehicle and cell density. The flow characteristics of suspensions with different cell volume fractions remains <0.1 Re (Fig. 3E). A uniform laminar flow therefore characterizes the passage of even very high-density cell suspensions through the needle barrel. However, it is noteworthy that higher ejection speeds using PBS and pluronic with a small-bore needle (32G) increase Reynolds number >0.03. HTS maintained more steady flow characteristics across different needle sizes and cell volume fractions.

### Cellular fraction in suspension affects shear stress

**Figure 4:**
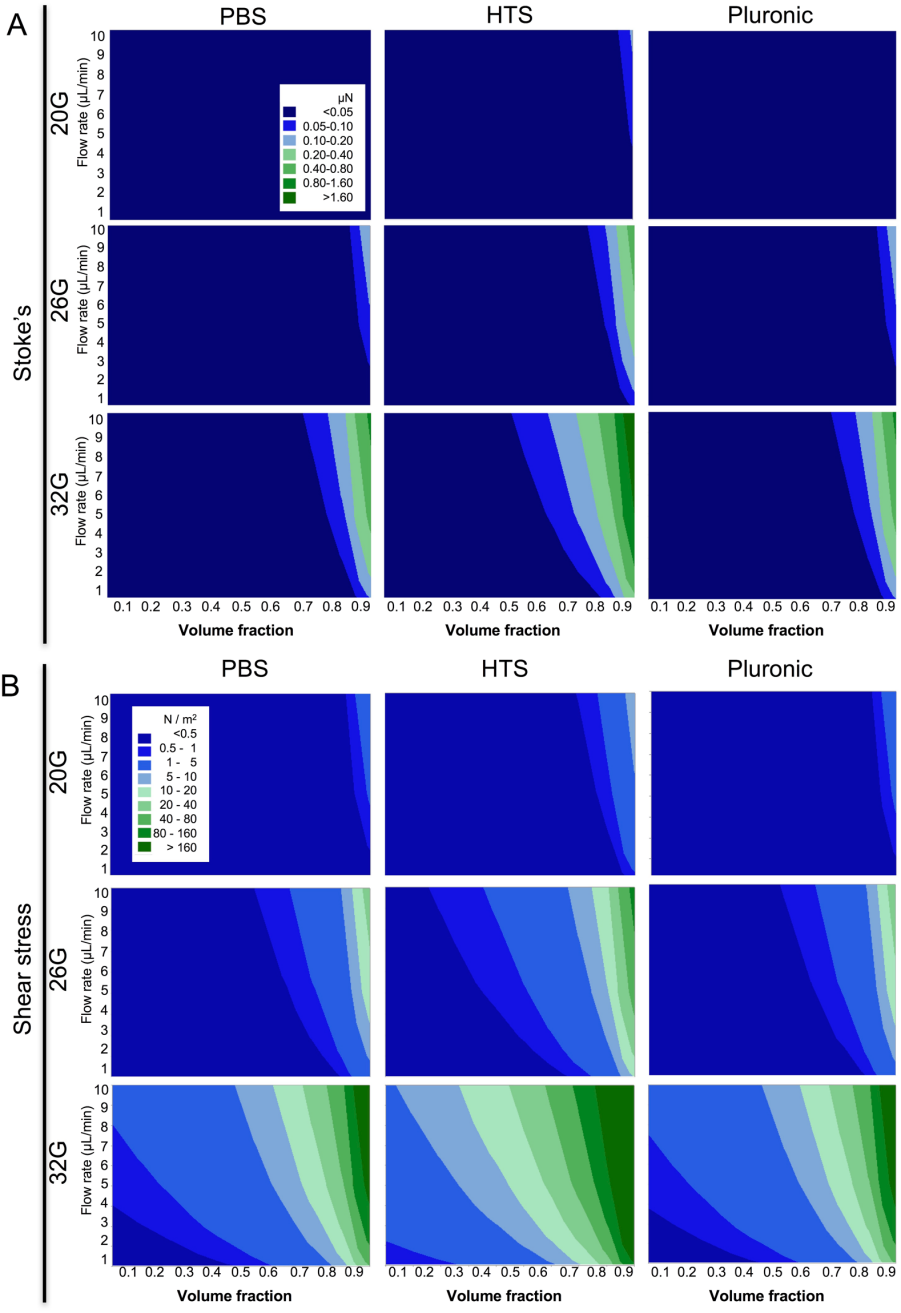
Contour plots for Stoke’s drag force and shear stress influencing cells in suspension. (**A**) The drag force (μN) in a 20G and 26G needle is neglible for all practical cell suspension (<0.6 volume fraction). The smaller dimater of the 32G needle, as well as the higher viscosity of HTS, exert greated drag force on cells than other conditions, but these are still very low and unlikely to influence cells or their sedimentation. (**B**) Shear stress (N/m^2^) is increased with smaller diameter needles, higher viscosity and flow rate. A 32G needle with a high cell volume fraction in HTS therefore will be exposed to the highest shear rate. However, the use of a 26G needle significantly reduces this shear stress.

The cells’ speed of sedimentation is also influenced by the frictional drag force (*F*_*d*_), i.e. fluid resistance, that the vehicle exerts on the cells and flow rate of the vehicles. In laminar flow conditions, *F*_*d*_ can be described using Stoke’s law. In most cases, *F*_*d*_ is negligible indicating that gravity will exert a sedimentation effect on cells (Fig. 4A). Indeed, using a 20G needle. virtually no *F*_*d*_ is exerted on cells. Only using a 32G needle, *F*_*d*_ is apparent at high cell densities and flow rates. In the case of PBS and pluronic, no *F*_*d*_ is apparent at volume fractions that could be prepared with cells (i.e. <60%). Only with HTS some *F*_*d*_ is exerted on cells at volume fractions >50% and a flow rate >5 μL/min. The influence of *F*_*d*_ is therefore negligible on the sedimentation of cells in suspensions.

Increasing cell fractions lead an increase in cell suspension viscosity. This change in viscosity also affects shear stress. Although a 20G needle provides almost homogenous shear stress conditions across all cell volume fractions, suspension vehicles and flow rates, as well as narrowing of needle diameter produce greater shear stress (Fig. 4B). For 26G needles, PBS and pluronic remain below 0.5 N/m^2^ for 60% volume fractions up to a flow rate of 5 μL/min. HTS cell suspensions undergo greater shear stress with a 60% volume fraction suspension at 1 μL/min staying under the 0.5 N/m^2^ threshold. A 5 μL/min flow rate at this level of shear stress can be achieved using a volume fraction of 30%. The use of a 32G needle is very restrictive for shear stress to remain under 0.5 N/m^2^. These results highlight the importance of accounting for cell volume fraction, flow rate and vehicle viscosity to ensure that damage to cells during the injection process is minimized.

### Slow flow rate improves viability after passage through narrow bore needles

**Figure 5:**
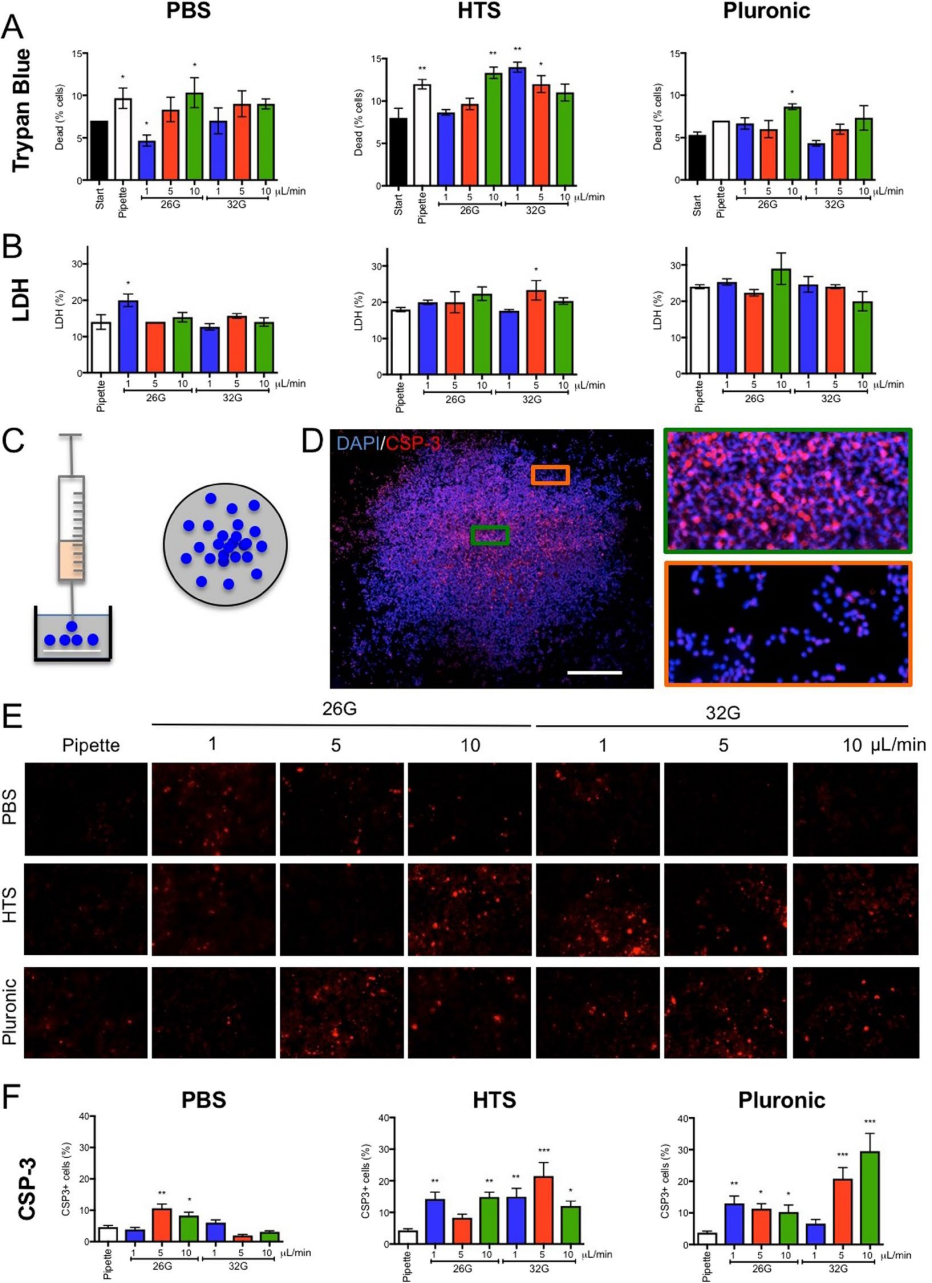
Cell viability, cell membrane damage and apoptosis after syringe-needle ejection. (**A**) Viability measurements using trypan blue were used to determine the percentage of dead cells upon preparation of cell suspensions (20% volume fraction, 50,000 cells/μL) with different suspension vehicles, as well as after ejection. (**B**) To determine if the ejection procedure induced cell membrane damage, lactate dehydrogenase (LDH) was measured 24 hours after ejection and compared to pipetting of cells. (**C**) To model *in vitro* the injection procedure, cell suspension were placed as a deposit on a cover slip with gentile agitation to spread cells. (**D**) This *in vitro* injection model produce a greater cell density at the centre of the coverlsip with a lower cell density at the edge of the deposit. Caspase-3 (CSP-3) immunocytochemistry was used to define cells undergoing apoptosis. A greater degree of apoptosis was evident after 24 hours at the core of the deposit compared to the corona. (**E**) The number of CSP-3+ cells were counted for each ejection. Very few apoptotic cells were evident in the PBS condition compared to HTS and Pluronic. It was further evident that a higher flow rate and thinner needle increased the number of apoptotic cells. (**F**) Ejection of cells using pluronic as suspension vehicle through a 32G needle at a high flow rate produced the highest proportion of apoptotic cells, whereas a slow ejection using a larger needle and PBS had minimal impact.

To determine the impact of cells’ passaging through a syringe and narrow bore needle, cell viability was measured straight after ejection using the different suspension vehicles at different flow rates (Fig. 5A). A slower flow rate reduced the number of dead cells in the ejectate, with 1 μL/min inducing the least dead cells (~5%). Pluronic as a suspension vehicle resulted only in a minor increase in cell death at 10 μL/min flow rate, with all conditions having <10% dead cells. In contrast, HTS exhibited the highest level of cell death with dead cells exceeding 10% by using a 32G needle. Slow flow rates and pluronic as suspension vehicle hence provide favorable conditions to preserve cell viability.

As acute viability might be an insufficient predictor of cellular effects caused by passage through a narrow bore needle, cell membrane damage was also investigated 24 hours after ejection (Fig. 5B). Surprisingly little influence of flow rate or needle diameter was observed on cell membrane damage. In contrast, increased cell damage was observed with HTS and pluronic in comparison to PBS, suggesting that more viscous fluids might exert mechanical damage to cells that can lead to a delayed effect on cell survival.

To determine the effect of ejection parameters on cell apoptosis, cells were ejected by mimicking the injection procedure onto a coverslip (Fig. 5C). This produced a dense core area on the coverslip with more diffusely spread cells at the edge of the coverslip (Fig. 5D), reflecting the *in situ* condition where a cell plug is observed at the deposit site with a more diffuse cell distribution due to migration into the surrounding tissue. Using this approach, the number of cells positive for caspase-3 were counted in relation to the total number of cells present to define how many cells were undergoing apoptosis 24 hours post-ejection (Fig. 5E). PBS produces the lowest number of apoptotic cells (<10%). HTS and pluronic produced significantly higher proportions of apoptotic cells ranging between 7–28% (Fig. 5F). A smaller diameter bore at higher speeds of 5 and 10 μL/min for HTS and pluronic produced more apoptotic cells. Higher flow rates with viscous vehicles can therefore results in a delayed cell death.

### Flow rate affects neuronal differentiation of hNSCs

**Figure 6:**
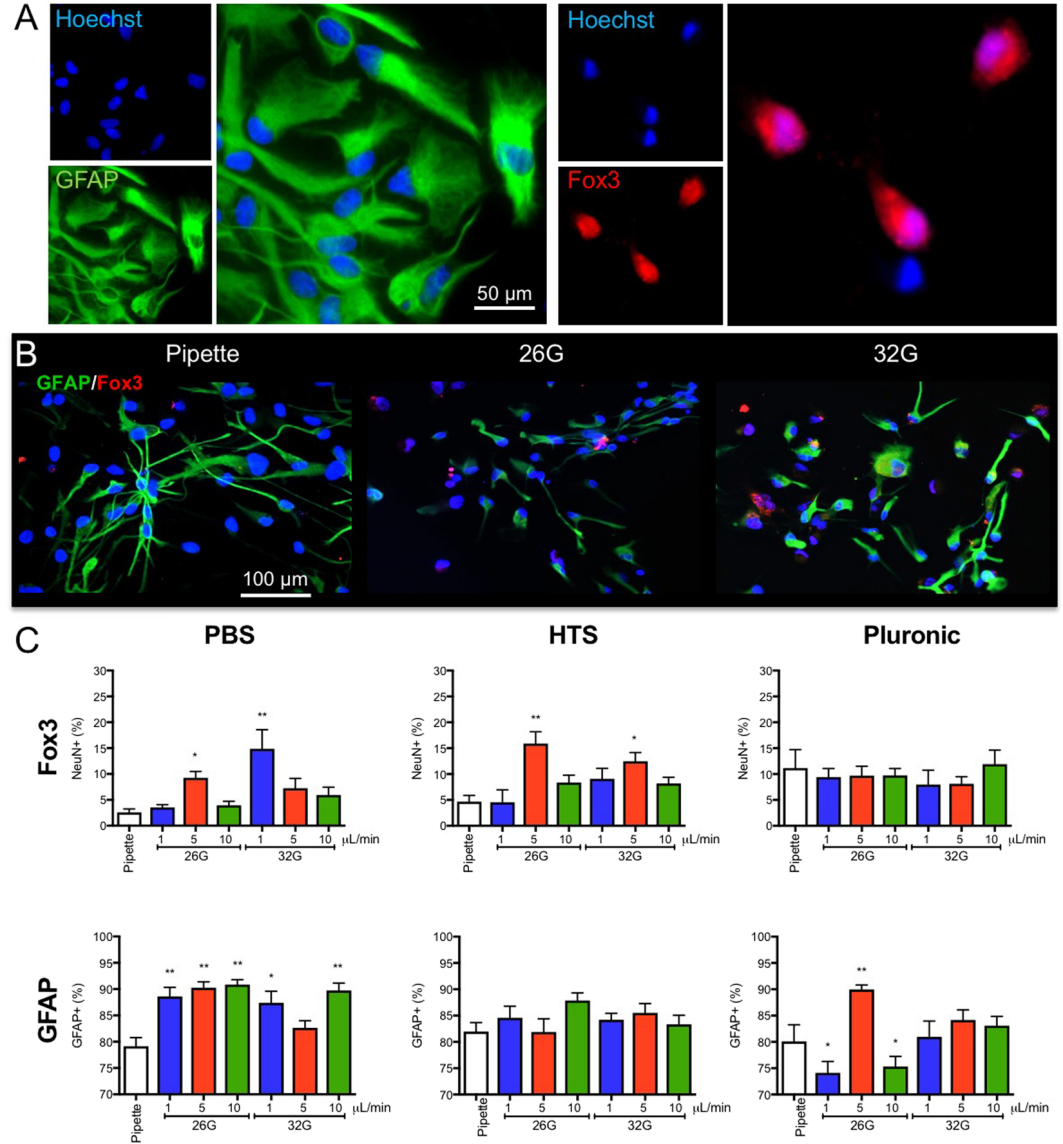
Ejection conditions affect neuronal and astrocytic differentiation of NSCs. (**A**) Neural stem cells differentiate into both astrocytic (GFAP+) and neuronal cells (Fox3) with distinct morphologies. Typically astrocytes are found in dense cell clusters, whereas neurons were more common in less densely populated areas. (**B**) Astrocytic and neuronal differentiation of NSCs after ejection from a pipette, 26G or 32G needle, at a 20% volume fraction (50,000 cells/μL) revealed both morphological, as well as phenotypic differences after 7 days of differentiation. (**C**) Ejection parameters influenced neuronal and astrocytic differentiation of NSCs compared to pipette only ejection. Especially PBS resulted in an overall increased astrocytic differentiation, while at 5 and 1 μL/min ejection using a 26G and 32G needles respectively produced more neuronal differentiation. HTS reduced the impact of ejection on cell differentiation, although a 5 μl/min flow rate using a 26G needle increased neuronal differentiation. Neuronal differentiation was also maintained constant with pluronic as suspension vehicle, but significant shift in astrocytic differentiation were evident.

A key property of hNSCs for cell transplantation studies is their appropriate phenotypic differentiation (Fig. 6A), which could be affected by biomechanical forces impacting cells during their passage through a syringe and narrow bore needle (Fig. 6B). Pluronic did not affect neuronal differentiation at any flow rate, whereas HTS (10%) and PBS (7%) both saw an increase in Fox3 + cells after 7 days of differentiation with a flow rate of 5 μL/min through a 26G needle (Fig. 6C). Increases were also evident with 32G needles at a speed of 1 μL/min for PBS (12%) and 5 μL/min for HTS (8%). For astrocytic differentiation, a significant increase (~9%) in GFAP + cells was evident for PBS with a 26G needle, whereas a ~10% increase was observed for the pluornic suspension with a 26G needle and flow rate of 5 μL/min. These results indicate that interactions between barrel diameter and vehicle viscosity lead to different shear stressors that can differentially affect cell differentiation. Understanding these interactions will hence improve our ability to minimize bio physical effects on cell survival and differentiation.

## Discussion

The therapeutic efficacy of cell transplantation is dependent on the delivery of viable cells through a narrow bore syringe-needle without affecting the cells’ mechanism of action or potential for differentiation^1^. We here demonstrated that delivery can significantly impact cell viability, as well as differentiation. The suspension vehicle can dramatically influence the biomechanics of the ejection procedure, but needle size and speed of ejection can be adjusted to minimize biological effects.

### Biomechanical effects of cell ejection through narrow bore needles

During the uptake and ejection of cells in suspension, cells are exposed to pressure, as well as shear stress, that can affect their functions^23,24^. Normal intracranial pressure is considered to range between 0.93–1.99 kPA with measurements >3.33 kPA typically requiring treatment to mitigate adverse events^14^. We here demonstrated that all tested conditions exceed normal intracranial pressure by at least 50%. Smaller needle sizes and slow ejection speeds exposed cells to the highest pressure. Even the pressure inside the 20G needle exceeded treatment requiring intracranial pressure by more than 15x. Cells are hence exposed to high levels of pressure during the ejection process, although this is only for a short amount of time. Ideally, gastight needle-syringe preparations are used, as these have a single sized barrel for needle and syringe, avoiding pressure points that could adversely affect cells.

Interaction of cells with the barrel walls further exposes them to shear stress that can lead to membrane damage leading to cell lysis or apoptosis. Higher viscosities lead to higher shear stress. This was evident in the case of HTS, which produced the highest level of shear stress (4 N/m^2^ = 4 Pa), whereas cells suspended in PBS experienced the least shear stress (0.5 N/m^2^). A smaller needle diameter also induces more shear stress, again indicating that a 32G needle is very narrow for cell injection (<5 cells diameter). Although exposure to shear stress is short during the uptake and ejection procedure, a prolonged (12 hours) shear stress of 1 N/m^2^ (10 dyn/cm^2^) has been associated with increased cell death^19^, as well as an increased influx of Ca^2+^ ions into neurons and astrocytes^25^. In contrast, 0.5 N/m^2^ had no effect on cell viability^19^.

Although endothelial cells are constantly exposed to shear stress and can be expected to be more tolerant of these forces^23^, NSCs typically do not experience shear stress. Indeed, shear stress is the main driver of neuronal damage in traumatic brain injury^26^. Immediate cell death after ejection was minimal here, suggesting that only a small percentage of cells were lysed by ejection. HTS with a 32G needle produced the highest shear stress and resulted in 10% cell death. A slower ejection, however, reduced this and the use of a larger 26G needle further reduced cell death to less than 5%. A delayed effect was evident here with more viscous suspension vehicles, such as HTS and faster ejection speed producing up to 28% of apoptotic cells within 24 hrs post-ejection. Acute shear stress therefore only affected a small proportion of cells that are likely to be lysed during the ejection and detectable using a viability assay. Although acute cell membrane damage is low, a substantial delayed cell death can potentially reduce the survival of cells after transplantation.

### Sedimentation and ejection flow characteristics

Needle/transcatheter induced biomechanical forces have also been reported to influence the therapeutic potential of mesenchymal stem cells^9,11–13^. Mechanotransduction can play a major role in cell differentiation^27^ that influences therapeutic efficacy. We here established that neuronal differentiation was increased with a flow rate of 5 μL/min in a 26G needle for both PBS and HTS, but not pluronic. A 32G needle with a flow rate of 1 μL/min using PBS also achieved an increase in neuronal differentiation, indicating that specific combinations of shear stress and pressure can exert substantial shifts in cell behavior. A stiffness of 0.7 kPa promoted neuronal maturation, but this brain-like stiffness (0.3–0.8 kPa) does not affect the proportion of neuron subtypes^28^. Others have demonstrated that neurite extension in neuronal differentiation is decreased on stiffer (4.2–7.9 kPA) compared to less stiffer substrates (0.1–0.8 kPa)^29^, indicating the influence of biomechanical forces on differentiation, as well as neuronal function. PBS and pluronic here also showed a shift in astroglia differentiation, which has been thought to be associated with higher stiffness/shear stress^30^. Although these biomechanical forces exerting effects on neuronal differentiation cannot be completely eliminated from the delivery process, it is nevertheless important to minimize these if neuronal differentiation is the pivotal mechanism of recovery.

As ejection pressure and shear stress are dependent on the viscosity of the material passing through the barrel, it is critical to note that the cellular fraction in a cell suspension affects viscosity and hence influences these parameters and their influence on cell behavior. Higher cell volume fraction increases viscosity, which is consistent with other reports demonstrating rheological changes in cell suspension with varying cell concentration^31^. Suspension vehicle also affects viscosity by defining the space in between cells and guiding their potential interaction with each other. Lower viscosity vehicles, such as PBS, can support higher cell volume fractions, while maintaining a lower viscosity. Higher viscosity materials, such as HTS, in contrast, might be more favorable for lower cell volume fraction suspensions, as more viscous fluids maintain more consistent single cell suspensions by limiting agglomeration of cells^32^. Although uniform laminar streamline flow is retained, with increasing viscosity of the suspension, shear stress is dramatically increased. Cell fraction has a dramatic effect on the biomechanical properties of suspensions. To keep shear stress under 0.5 N/m^2^ requires cell suspension containing <40% cell volume fraction and a flow rate of 5 μL/min. These are important considerations for intracerebral cell transplantation.

Cell volume fraction also affects sedimentation/settling of cells^6^. Higher cell volume fractions reduce sedimentation by increasing the suspension viscosity. A key difference for intracerebral injections in a clinical and preclinical setting is that animals are typically implanted using a vertical plane (90°) to deliver cells into the brain, whereas neurosurgeons will use a horizontal plane (0°) to administer cells. We here confirmed that sedimentation in a cannula is minimal along the horizontal plane (<5 μm/s), but there is significant sedimentation along the vertical plane (>80 μm/s). The sedimentation is further influenced by cell density and viscosity of the material. Faster sedimentation will lead to an uneven payload delivery, but can also lead to the formation of a plug that blocks the ejection^33^. In this case, additional pressure is required and displacement of the plug can lead to a bolus ejection, rather than convection-enhanced delivery. Careful consideration of the biomechanics of the cell suspension and measurement of applied force during delivery can potentially mitigate these delivery issues.

### Implications for intracerebral cell implantation

To ensure consistency between measures, ejections here were preformed into a fluid *in vitro* to model the *in vivo* process in the absence of variables that could affect measurements, such as different tissue types, as well as variability in the placement of and potential blockage of cannulas^34^. Ejection into a fluid allows the ejectate to freely dissipate, whereas injection into a tissue that behaves like a solid will form a cell deposit that increases resistance and ejection pressure. Haptic feedback during manual injection can allow surgeons to adjust the applied force to compensate for a tissue-based build-up of resistance to the injection. However, a comparison of injection pressure in a simulated peripheral nerve block administration by anesthesiologist found that the perception of applied force and rate of injection varied widely and generally was too high^35^. Defining safe ejection rates and monitoring applied force are therefore important advances that facilitate a robust implementation of convection-enhanced delivery^36^. The interstitial pressure inside the tissue can also lead to a reflux phenomenon^37,38^ that can lead to an expulsion of transplanted cells from the brain^39^. Further *in vivo* studies will be required to determine how ejection into tissue will affect pressure in the syringe-needle, as well as the dispersion of cells from the injection site.

In addition to the density and viscosity of the material, the volume, as well as the speed of injection, are also likely to influence potentially adverse events. *In vivo* injection studies therefore need to determine appropriate volumes and injection speeds to define the dispersion area of the injectate^40^. Damaged tissue, as in the case of stroke, is likely to be more permeable than intact tissue^5^. Nevertheless, coverage of large areas of damage might require several injection sites and tracts to ensure an appropriate coverage of transplanted cells^41–43^. Especially in a clinical setting larger anatomical structures will require extensive areas for cells to spread. Needle length is also typically longer (15.2 cm) and hence will expose cells to shear stress for longer periods^10,44^. However, 20G needles are commonly used clinically, which will mitigate shear stress and injection pressure compared to small rodents using 26G needles. These differences further underpin key biomechanical variables in cell delivery in a clinical and small rodent setting that could have implications for the biological action of implanted cells^1,45^. Establishing efficient injection protocols therefore is likely to require both small and large animal models^45–47^.

## Conclusion

The therapeutic success of cell therapy is dependent on the efficient and safe delivery of cells to the brain. We here demonstrated the importance of considering the biomechanical variables that influence this process. A shear stress <0.5 N/m^2^ is exerting minimum impact on cell survival and differentiation. Cell volume fractions at <40% delivered with a 26G or larger needle at <5 μL/min will achieve this using PBS, HTS or pluronic. Smaller 32G needles are unfavorable for cell injections. For vertical injections, lower (<20%) cell volume fractions will be best formulated with more viscous vehicles (>100 cP combined), whereas higher cell volume fractions (>20%) need less viscous vehicle to mitigate shear stress, as well as sedimentation during the ejection process. A horizontal injection, as commonly used in clinical settings, reduces sedimentation and requires less consideration for vehicle viscosity. The delivery process is hence a crucial step in the intracerebral implantation of cells that requires the definition of appropriate parameters to ensure the efficacious translation of this promising therapy.

## Electronic supplementary material

Supplementary Figures
