# Supplementary material for: Ex vivo biomechanical characterization of syringe-needle ejections for intracerebral cell delivery
Source: Sci Rep. 2018 Jun 15;8:9194. doi: 10.1038/s41598-018-27568-x (PMC6004017; doi:10.1038/s41598-018-27568-x)
Supplement: Supplementary file 1 — Supplementary Figures [file 41598_2018_27568_MOESM1_ESM.pdf]

Ex vivo biomechanical characterization of syringe-needle ejections for intracerebral cell delivery.

Brendon Wahlberg[1*], Harmanvir Ghuman[2,3*], Jessie R. Liu[2] & Michel Modo[1,2,3,4].

University of Pittsburgh, [1]Departments of Radiology and [2]Bioengineering, [3]McGowan Institute for Regenerative Medicine,
[4]Centre for Neural Basis of Cognition,
Pittsburgh, PA15203, USA

* both authors contributed equally

Corresponding Author:
Dr. Mike Modo
University of Pittsburgh
McGowan Institute for Regenerative Medicine
3025 East Carson St
Pittsburgh, PA 15203
USA
+1 (412) 383 7200
e-mail: mmm154@pitt.edu

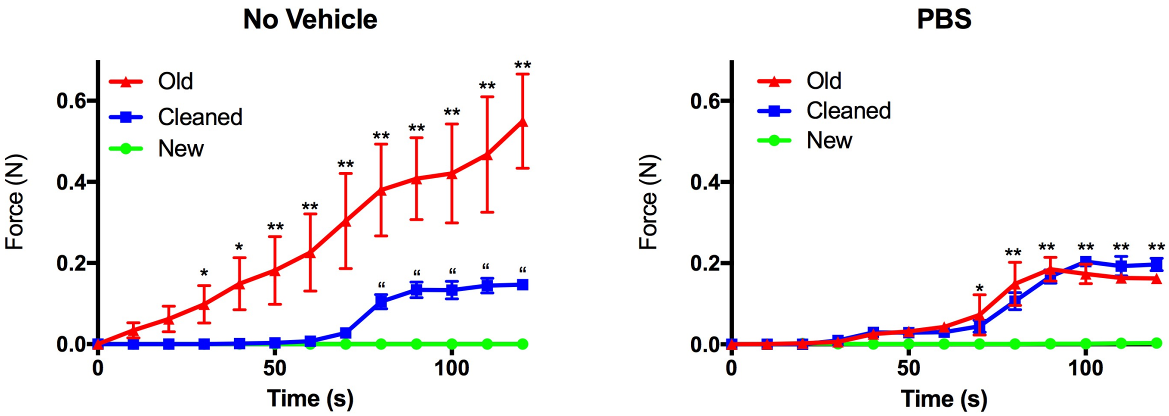

Supplementary Figure 1. Quality control for syringe use. To define operational criteria for the use of 50 µL Hamilton syringes, the force required to expell PBS was measured in an old, a cleaned and a new syringe. Old needles required significantly more force to move the plunger compared to a cleaned and new syringe. In the absence of a liquid this force in old syringes is even higher. We propose the use of a 0.2 N threshold without liquid to discard used syringes and apply a measure of quality control.

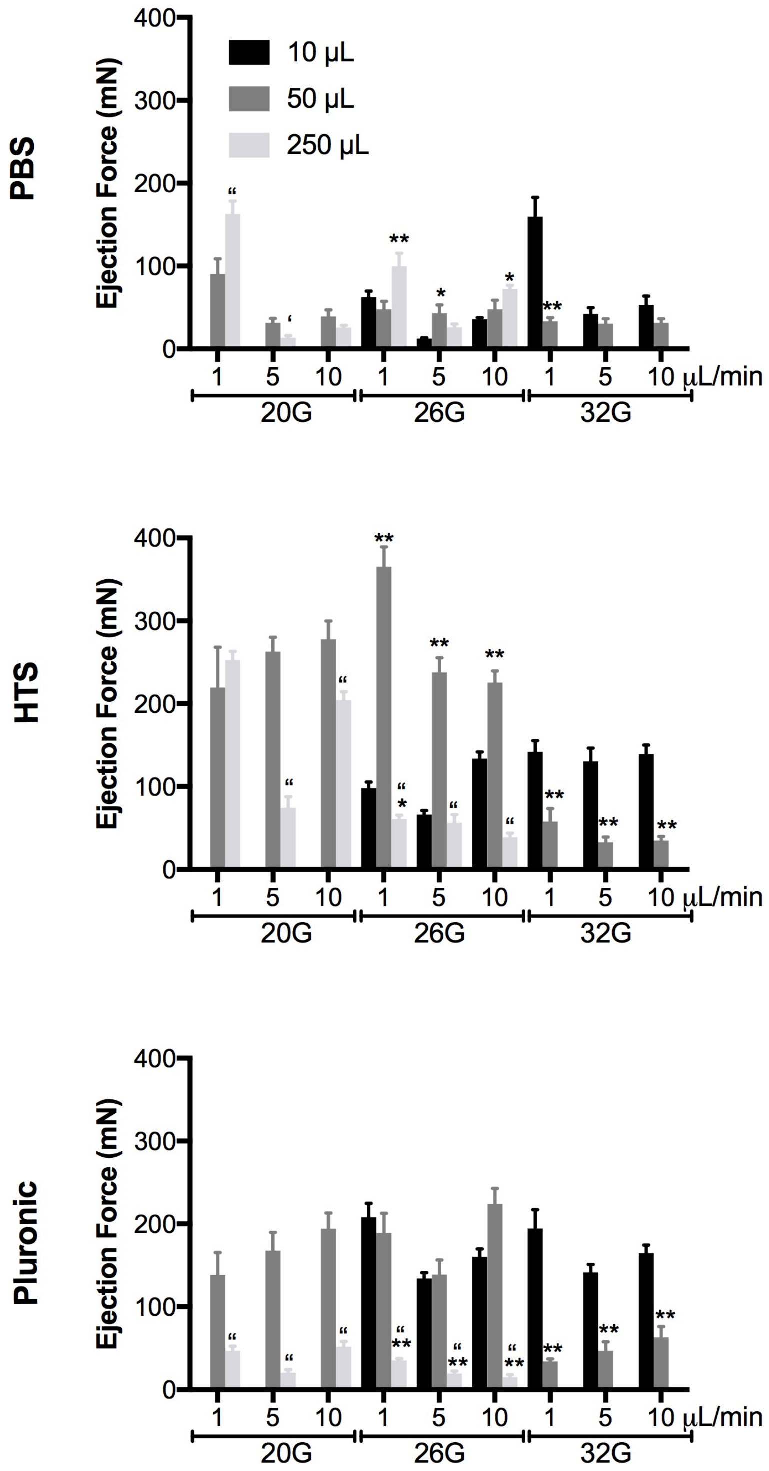

Supplementary Figure 2. Ejection force measurments. A miniature compression load cell was used to measure the maximum force required to eject 10 µL of vehicle under different ejection parameters. The maximum force measurements were then converted to ejection pressures by dividing the force by cross-sectional area of the syringe or needle.

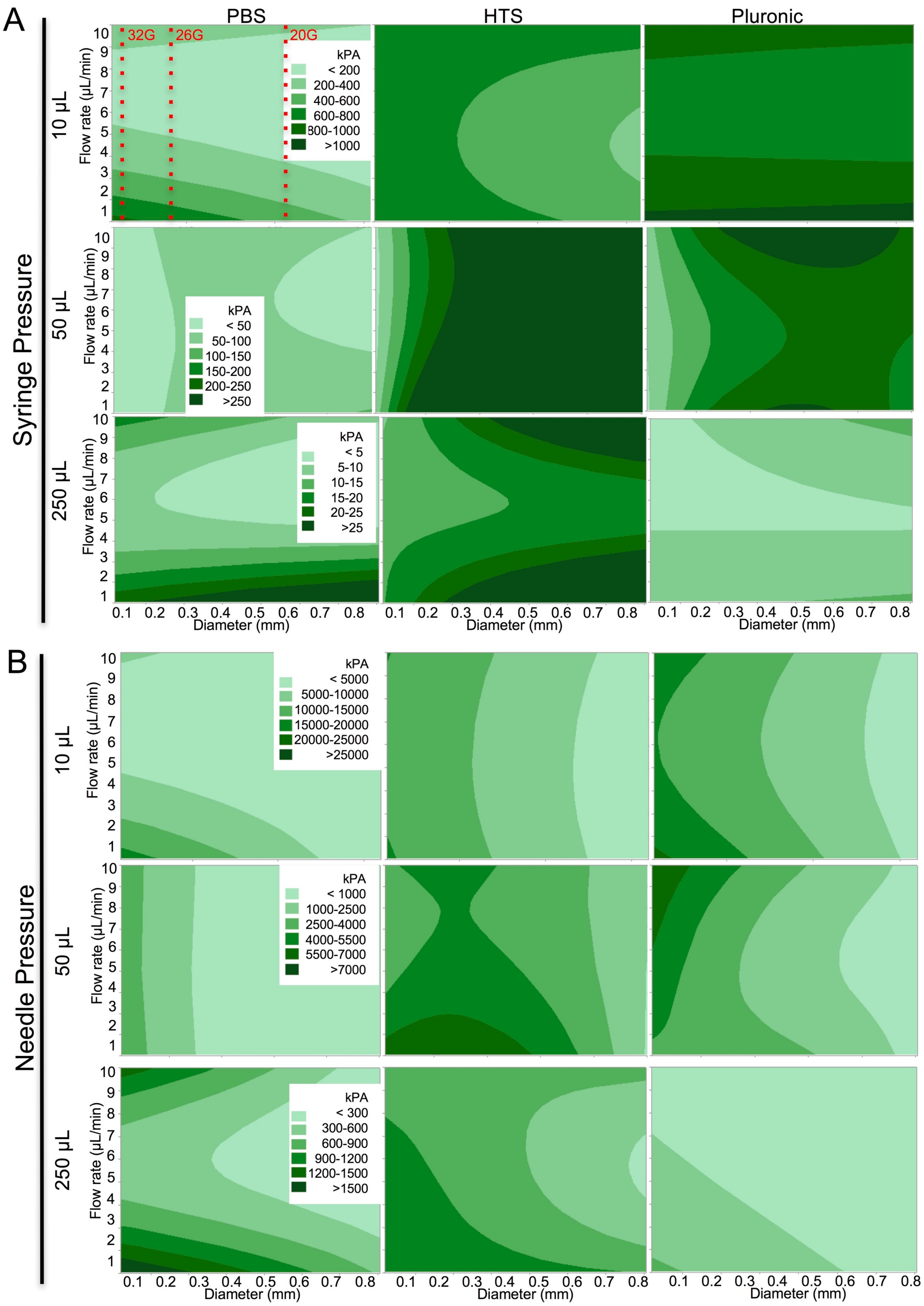

Supplementary Figure 3. Contour maps of ejection pressure. A. Ejection pressure (kPa) is lower with larger syringes and needles. Especially pluronic had low ejection pressure, whereas HTS requires higher ejection pressure. **B**. Inside needles, ejection pressure was much higher than in syringes due to the overall smaller bore diameter. A medium flow rate of 5 µL/min generally reduced ejection pressure.

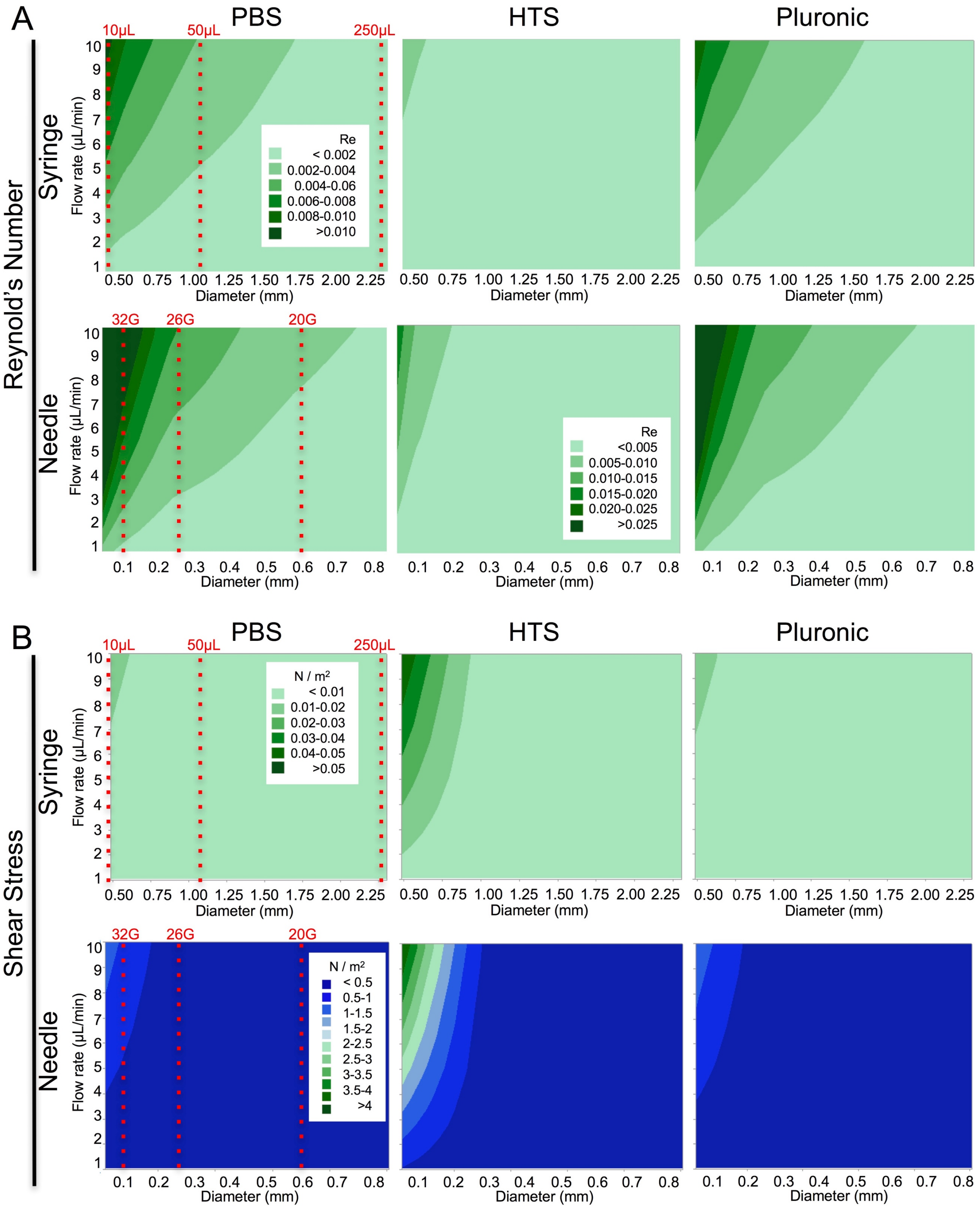

Supplementary Figure 4. Contour maps of Reynold's number and shear stress. A. Reynold's number (Re) increased through an interaction of an increasing flow rate and decreasing barrel diameter. Less viscous fluids resulted in higher Reynold's numbers, but all were Re <0.1 indication uniform laminar flow. **B**. Shear stress (N/m^2) in needles was much higher than in syringes. Increased shear stress was also determined by increased flow rate and a smaller diameter, but less viscous material produced less shear stress. HTS therefore had higher shear stress compared to PBS and pluronic.
